# Robust indexing for automatic data collection

**DOI:** 10.1107/S0021889804005874

**Published:** 2004-05-11

**Authors:** Nicholas K. Sauter, Ralf W. Grosse-Kunstleve, Paul D. Adams

**Affiliations:** a Department of Physical Biosciences, Lawrence Berkeley National Laboratory, 1 Cyclotron Road, Bldg 4R0230, Berkeley, CA 94720, USA

**Keywords:** indexing, autoindexing, data collection, computer programs, high-throughput automation, macromolecular crystallography

## Abstract

Improved methods for indexing diffraction patterns from macromolecular crystals are presented. The novel procedures include a more robust way to verify the position of the incident X-ray beam on the detector, an algorithm to verify that the deduced lattice basis is consistent with the observations, and an alternative approach to identify the metric symmetry of the lattice. These methods help to correct failures commonly experienced during indexing, and increase the overall success rate of the process. Rapid indexing, without the need for visual inspection, will play an important role as beamlines at synchrotron sources prepare for high-throughput automation.

## Abstract 2

Improved methods for indexing diffraction patterns from macromolecular crystals are presented. The novel procedures include a more robust way to verify the position of the incident X-ray beam on the detector, an algorithm to verify that the deduced lattice basis is consistent with the observations, and an alternative approach to identify the metric symmetry of the lattice.

## 1. Introduction

Large-scale macromolecular crystallography projects, including structural genomics efforts (Stevens *et al.*, 2001 ▶), are placing increasing demands on synchrotron beamline facilities worldwide. In response, new methods are being developed to increase efficiency and throughput. Many beamlines are now being equipped with sample-handling robots (Cohen *et al.*, 2002 ▶; Snell *et al.*, 2004 ▶), and new graphical user interfaces provide the experimentalist with flexible control over the data acquisition process (McPhillips *et al.*, 2002 ▶). Additional efforts are under way to provide a measure of automation to the subsequent stages of data reduction (Leslie *et al.*, 2002 ▶) and structure solution (Adams *et al.*, 2002 ▶; Brunzelle *et al.*, 2003 ▶).

Availability of convenient crystal handling at the beamline has enabled users to perform rapid screening experiments, wherein large numbers of similar crystals are briefly examined, with the best ones being identified for later collection of full data sets. Reasons to screen multiple samples include optimization of the cloning, expression, and purification techniques involved in protein production, determination of the most favorable crystallization and cryocooling conditions, and investigation of large numbers of macromolecule–ligand complexes. Furthermore, even crystals prepared under identical conditions can be heterogeneous. For each sample, the typical protocol involves acquiring a narrow (0.25–1.0° rotation) oscillation image at a standard distance, or if time permits, acquiring two images at rotation settings separated by 90°, to reject crystals with unacceptably high anisotropic diffraction. If control is fully automated, the data can be acquired in 1–2 min per sample.

Once acquired, images must be analyzed to determine if the diffraction pattern can be indexed. Indexing the crystal lattice and determining the likely Bravais symmetry permits the user to predict whether a given crystal can potentially yield a complete data set. This step must be completed in real time so that the user can select samples for further study. Presently there are several software packages available to assist with this (Pflugrath, 1997 ▶, 1999 ▶; Otwinowki & Minor, 1997 ▶; Leslie, 2001 ▶; Kabsch, 2001 ▶). Most include graphical user interfaces, which serve the important function of allowing the experimenter to visually confirm whether the diffraction pattern predicted by indexing matches the observations. If indexing is to be eliminated as the rate-limiting step, it must be very reliable even in the absence of this visual inspection. Indeed, it would be ideal if the indexing program ran in the background concurrently with data collection, so that results appear in close to real time as the images are acquired. Naive attempts to automate this process with shell scripts, however, revealed systematic problems with determining the beam center, lattice basis and symmetry. As we will show, these common problems can be detected algorithmically and corrected automatically. We have developed *LABELIT* (*Lawrence Berkeley Laboratory Indexing Toolbox*), a Python/C++ package capable of handling difficult indexing cases.

## 2. Computational methods

**Figure 1:**
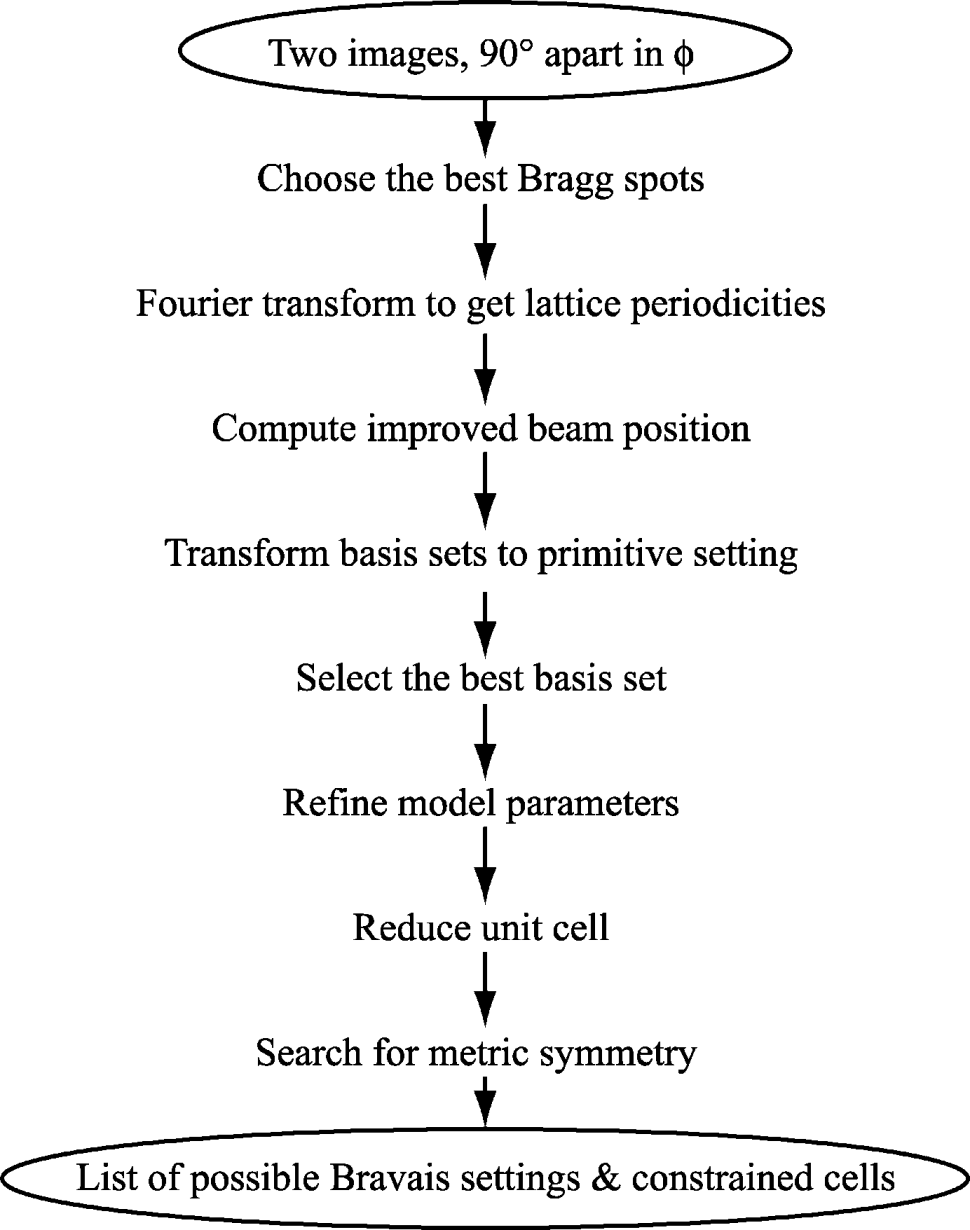
Overall indexing procedure.

The overall approach to indexing is summarized in the flow chart in Fig. 1 ▶. After the brightest Bragg spots are selected, spot positions are converted to reciprocal space, and the spot distribution is analyzed to detect periodicities corresponding to lattice spacings. The analysis of lattice spacings also gives sufficient information to improve the initial estimate for the direct-beam position. Three vectors showing significant periodicity in the spot distribution are chosen as the three basis vectors of the unit cell. A computational check is made to assure that the basis forms a primitive lattice rather than one in which some predicted spots are systematically absent. Once the model parameters are refined in a triclinic setting, tests are performed on the unit-cell dimensions to detect possible symmetry elements, which are then combined to produce a list of Bravais types consistent with the data. Cell refinement with symmetry-based restraints produces a final set of indexing solutions suitable for other procedures, such as determining the data collection strategy, or integrating the data set. Although Fig. 1 ▶ depicts data analysis running continuously through from start to finish, *LABELIT* will terminate and provide a report of difficulties if the input data are unsuitable for processing at any step.

### 2.1. Choice of candidate Bragg reflections for indexing

In order to facilitate indexing, an effort is made to select candidate spots that are most likely to be Bragg reflections. The algorithm, *DISTL*, is described in detail elsewhere (Zhang *et al.*, 2004 ▶). Of all local maxima on the image, only those with a peak height higher than a cutoff multiple of the local background noise are considered, thus eliminating weak reflections. The image is then divided into very thin concentric shells centered around the direct-beam position, and the pixel intensity distribution is examined to eliminate ice or powder rings. Filters are then applied based on spot size, intensity and shape. Candidate Bragg reflections are expected to have a unimodal distribution of pixel intensities; that is, a single peak rather than a group of closely associated local maxima. Therefore, spots with more than two local maxima are rejected; those with two are permitted because some medium-intensity spots have bimodal distributions due to statistical noise. Also, pairs of spots are rejected when they are separated by less than 1.2 diameters, assuring that artifacts of crystal splitting do not degrade the indexing process. The upper resolution limit is chosen conservatively (Method 2 of Zhang *et al.*, 2004 ▶) to avoid reflections at very high diffraction angles, which might cause indexing to fail (if the unit-cell dimensions are large enough, high-resolution spots can overlap, making it difficult to assign accurate Miller indices). If fewer than 40 good spots on any image remain, no attempt is made to index the lattice. Ideally, 300 spots with the highest signal-to-noise ratios are chosen on each image.

Ignoring the weakly diffracting Bragg reflections poses a potential problem if there is pseudo-centering in the lattice, which creates systematically weak reflections that can be overlooked. Unfortunately, it is not possible to correct this situation simply by including all weak reflections, since the indexing process would likely be overwhelmed by experimental noise. We favor the approach of initially indexing with the brightest Bragg reflections, and then performing a targeted search for systematically weak spots if the deduced lattice is centered. Although we have not implemented this yet, we plan to automate this process with *LABELIT* in the near future.

### 2.2. Detection by Fourier analysis of likely basis vectors

The crystal lattice is deduced starting with the set of *N* candidate Bragg spots determined according to §2.1. At the outset the position of each spot on the detector is measured. The rotational setting ϕ at which the spot satisfies the reflection conditions is not known accurately, but is taken here to be the mid-value of the oscillation range. These positional and angular data are then used to derive the reciprocal-space position **x** for each spot, following the conventions of Rossmann (1979 ▶). In cases using images at two different rotation settings (usually 90° apart in ϕ, where ϕ is the goniometer rotation), the respective lists of reciprocal-space vectors are merged. It should be noted that merging the lists in this way implicitly ignores the possibility that spots at the same detector coordinates on adjacent images might be partial slices through the same Bragg peak. We avoid this problem by choosing not to index pairs of images that are adjacent or separated by less than 4° in ϕ.

**Figure 2:**
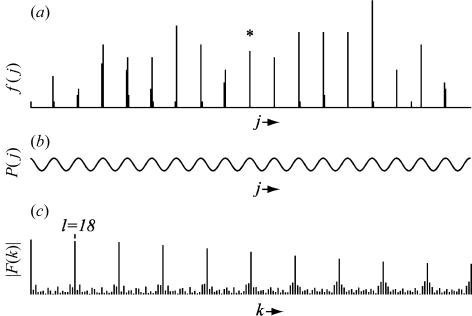
Analysis of observed Bragg reflections projected in reciprocal space onto a direction later determined to be the **a** axis of the sample’s orthorhombic unit cell. Panel (*a*) shows the histogram *f*(*j*) of reflections projected onto this axis, while panel (*c*) gives its power spectrum. The peak at *l* = 18 corresponds to a lattice periodicity along this axis of 36.4 Å. The single Fourier coefficient *F*(18) can be used [equation (5)] to create an approximate model for *f*(*j*), shown in panel (*b*). Note that the projection of the true beam position onto the axis, indicated by an asterisk (*), corresponds exactly to a crest in this model. Panels (*a*) and (*c*) correspond to Figs. 1 and 2 of Steller *et al.* (1997 ▶).

The procedure of Steller *et al.* (1997 ▶) is then used to determine which unit directions **t** are likely to form basis vectors for the periodic crystal lattice. Although we use the published method exactly, it is summarized here to provide a foundation for the following section. For every direction **t** chosen from a large set evenly spaced within the hemisphere, the projections *p* are calculated for all observed reciprocal-space points **x** onto **t**, 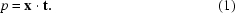
The range of *p* values is divided into *m* bins, each having an appropriately granular width Δ*p*. This allows us to construct a reciprocal-space frequency series *f*(*j*), giving the number of observed projections in the *j*th interval. Peaks in this series (Fig. 2 ▶ *a*) suggest the locations of possible reciprocal-lattice planes perpendicular to **t**. To find out how well the observed reciprocal-space points are described by periodic planes, one can take the discrete Fourier transform 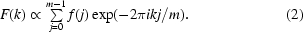
Peaks in the power spectrum |*F*(*k*)| correspond to strong periodicities along **t** (Fig. 2 ▶ *c*). In particular, the first and largest peak at *k = l* (not counting the peak at *k* = 0) is used to quantify the periodicity. After compiling a short list of **t** directions having the largest values of |*F*(*l*)|, each of these directions is refined with a fine grid search to maximize the |*F*(*l*)| value. Finally, directions that are collinear duplicates are rejected and the list is resorted, giving a set of ∼20 candidate directions {**t**}. Each candidate represents a possible unit-cell basis vector with real-space length 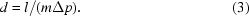
Only three directions are eventually chosen as true basis vectors (§2.4). The other directions in the set represent either linear combinations of the true basis vectors, lattice vectors due to the presence of a second crystal, or false periodicities.

### 2.3. A fast grid search to improve the direct-beam position

Since the direct X-ray beam often cannot be directly observed on the diffraction image, its position must be determined independently. This raises the possibility of systematic error in one’s prior belief about the beam position. If the prior belief is inaccurate, this will be reflected in the calculated reciprocal-space coordinates **x** of each observed Bragg reflection (Rossmann, 1979 ▶) and this will in turn compromise the autoindexing procedure. However, it is possible to use the Fourier coefficients *F*(*k*) derived from the given X-ray beam position to infer a better estimate of the true beam position. As will be described in detail in §3.1, a better estimate can be derived even if the input beam position differs from the true beam position by more than the smallest inter-spot spacing.

Recall that in general the frequency series *f*(*j*) may be recovered by the backwards Fourier transform 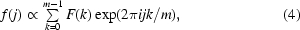
where the complex coefficients *F*(*k*) exhibit Hermitian symmetry, *F*(*k*) = *F*(*m* − *k*)*. Assume now that a particular direction **t**_0_ 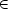 {**t**} is a basis vector for the unit cell, and therefore exhibits strong periodic groupings when the reciprocal-space points are considered in projection (Fig. 2 ▶ *a*). The essential information regarding this periodicity can be effectively modelled with the single Fourier coefficient *F*(*l*). By replacing the Fourier sum in equation (4) with a single term where *k* = *l*, *f*(*j*) can be approximated with the simplified expression 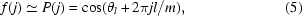
where θ_*l*_ is the polar angle in the conventional representation *F*(*l*) ≡ 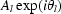.

As expected, crests in the sinusoidal plot of equation (5) (Fig. 2 ▶ *b*) closely correlate with the projections of observed reciprocal-lattice points in Fig. 2 ▶(*a*). Since the true beam position coincides with a reciprocal-lattice plane at **x** = 0, it will fall on one of the crests. This implies that *P*(*j*) in equation (5) can be viewed as giving the un-normalized conditional probability (in the Bayesian sense) that the true beam center projects onto direction **t**_0_ at interval *j*, given the prior belief about the beam center position.

**Figure 3:**
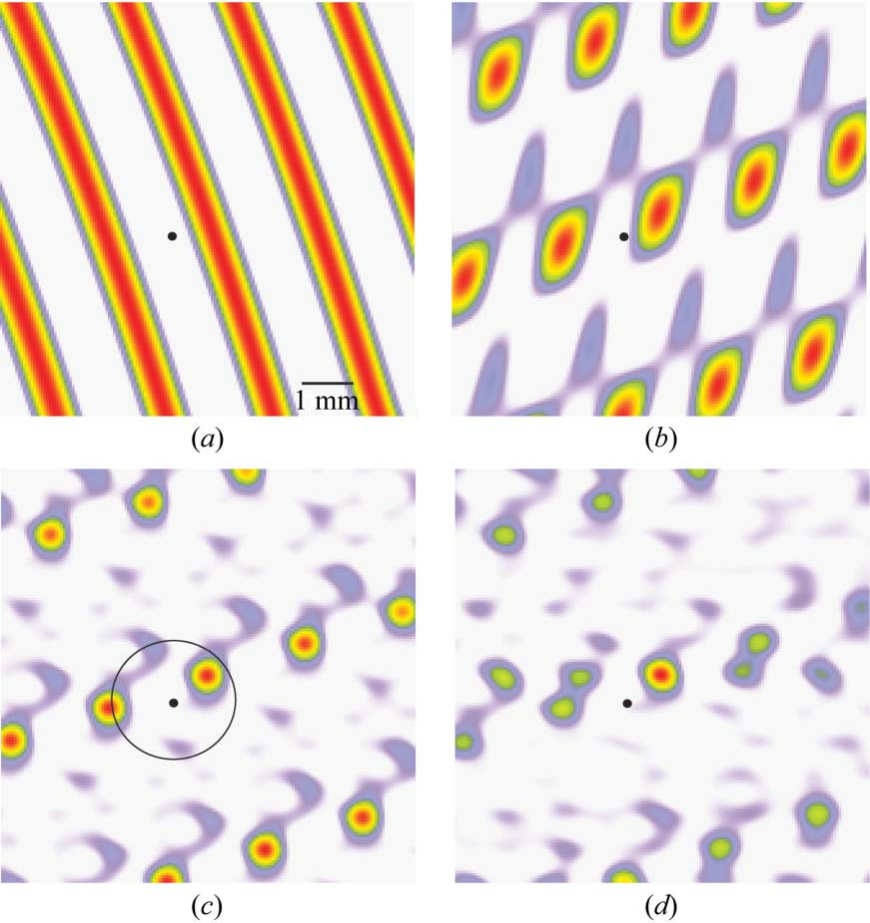
Map of a small portion of the imaging detector centered on the true beam position, depicting conditional probability contours for the location of the direct beam, given an input beam position (black dot). This particular input beam position is 0.8 mm from the true value. Probabilities were determined using the projections of reciprocal-space points from one image onto (*a*) the **b** axis, (*b*) all three unit-cell axes, and (*c*) the top 20 directions identified by Fourier analysis. Panel (*d*) uses projections of reciprocal-space points from two images collected 90° apart in ϕ, onto the top 20 directions. The circle in panel (*c*) shows the largest search radius, 1.2 mm, which could have been used to find the true beam center given the input value. In panel (*d*) the largest useful search radius is at least 3.3 mm. The crystal is the same as used in Fig. 1 ▶, with *a* = 36, *b* = 65 and *c* = 84 Å. 1° oscillation exposures were collected with a detector distance of 130 mm and an X-ray wavelength of 1.0 Å.

The true beam center cannot be deduced from equation (5) alone. As depicted in Fig. 2 ▶(*b*), each of several crests could potentially correspond to the true beam. Moreover, even if a particular crest at interval *j* is singled out, an infinite number of possible beam positions in the laboratory frame would be associated with this interval. This situation is summarized in Fig. 3 ▶(*a*), a contour map showing the probability that a given pair of laboratory coordinates is the true beam center. However, if three or more linearly independent directions **t**_0_, **t**_1_, **t**_2_ 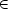 {**t**} are considered simultaneously, the possible laboratory coordinates of the true beam center can be constrained to a small set of points, as shown in Fig. 3 ▶(*b*). This contour plot has been computed as the sum of Fig. 3 ▶(*a*) and probability maps from two additional directions. For this example, the three directions **t**_0_, **t**_1_, **t**_2_ were selected to be the primitive axes of the unit cell.

In practice, one does not know which directions **t**_0_, **t**_1_, **t**_2_ will ultimately be chosen as basis vectors. Instead, probability fringes from all directions in the set {**t**} can be combined to help assure that the beam position is sufficiently overdetermined. An example of the resulting probability map is shown in Fig. 3 ▶(*c*). As will be discussed in §3.1, the search for the true beam position is confined to an area (indicated by a black circle in Fig. 3 ▶ *c*) within a radius *S* centered on the prior-belief beam center. Within this search radius, the true beam position is taken to be the peak position of the largest cluster, where clusters are ranked by integrated area. In the example illustrated, the true beam position is at the center of the panel.

Note in Fig. 3 ▶(*c*) that if the search radius *S* is made too large, there is the potential of misidentifying one of the other red peaks (not the one in the center) as the true beam position. This ambiguity disappears if data are available from two images collected at rotational settings 90° apart. If probability fringes from two images are combined, an unambiguous true beam position may be obtained over a much larger search radius (Fig. 3 ▶ *d*).

For use in the next section, the directions {**t**} must be improved based on the new estimated beam position. When two images are used, it is sufficient simply to re-refine each existing **t** using the fine grid search mentioned in §2.2. However, if only one image is used for indexing, the **t** directions are poorly determined at this point, and are unreliable when used for subsequent indexing steps. In this case, a completely new search for **t** directions is performed, with the new beam position as input.

### 2.4. Indexing the diffraction pattern

From the set {**t**}, three unit directions and associated real-space lengths [equation (3)] can now be chosen to form basis vectors for the unit cell: 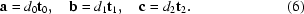
It is convenient to express this basis as the matrix of cell-axis components taken with respect to the orthogonal camera axes (Rossmann, 1979 ▶), calculated when ϕ = 0. Also, the inverse of this matrix gives the reciprocal-space components: 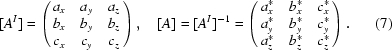
With these component matrices in hand, it is now possible to take the reciprocal-space coordinates of each observed Bragg spot, which were previously expressed in the reciprocal orthonormal basis (**x** in §2.2), and re-express them in terms of the reciprocal unit-cell basis vectors: 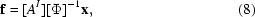
where [Φ] is a rotation matrix around the camera’s spindle corresponding to the ϕ setting of the particular image. Integer-valued Miller indices **h** are computed by rounding the real-valued components of **f**. It is important to realise that this simplistic method for calculating **h** does not account for the finite oscillation range Δϕ of each diffraction image, which tends to produce overlapping spots whose Miller index assignments are ambiguous. To guard against this, **f** is recalculated at each limit of the oscillation range (ϕ + Δϕ/2 and ϕ − Δϕ/2). If the same value of **h** is not produced in each case, the particular Bragg spot under consideration is disregarded for all subsequent calculations. Similarly, Bragg spots are ignored when they are too close to the rotation spindle for accurate evaluation.

### 2.5. Detection and correction of a non-primitive basis

**Figure 4:**
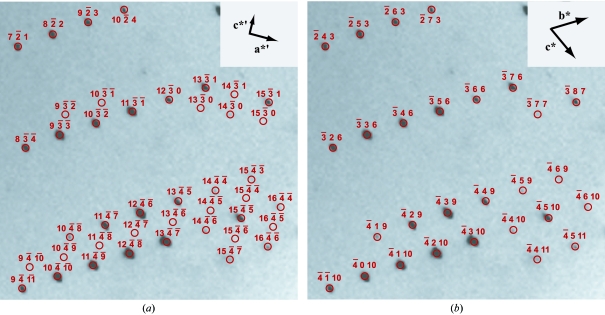
(*a*) Detail of a misindexed image, with incorrect reciprocal-space basis vectors a*′ and c*′ shown in the inset. Although all observed reflections are spanned by this basis, no reflections are observed when *h*′ + *l*′ is an odd number. (*b*) Corrected basis after application of the algorithm in §2.5. New basis vectors are chosen such that a* = b*′, b* = a*′ + c*′, c* = a*′ − c*′. The reader will note that the new Miller indices are given by *h* = *k*′, *k* = (*h*′ + *l*′)/2, *l* = (*h*′ − *l*′)/2. The matrix transforming the Miller indices is the inverse transpose of the matrix transforming the basis vectors.

As noted in §2.3, the set {**t**} is most likely to contain vectors that can form a primitive basis of the crystal lattice. However, it is also possible for {**t**} to contain directions that are linear combinations of primitive basis vectors, which should not be used to index the lattice. Fig. 4 ▶(*a*) illustrates a case where choosing a group of three highly ranked directions (*i.e.* directions with large |*F*(*l*)| values) leads to misindexing. The apparent reciprocal cell basis (Fig. 4 ▶ *a*, inset) predicts too many Bragg spots; further inspection reveals that reflections are only observed when *h* + *l* = 2*n*, where *n* is an integer. In general, the data must be scrutinized to find reflection conditions of the form 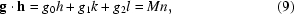
where *g*_0_, *g*_1_ and *g*_2_ are small integer coefficients and the modulus *M* is a small prime number, usually 2, 3 or 5. A transformation must then be applied to the incorrect basis [a*′, b*′, c*′] to produce the primitive one, 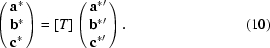
The matrix [*T*] must have integer coefficients and determinant *M*, but is otherwise not uniquely determined. We propose the following algorithm to enumerate the reflection conditions [**g**, *M*] and the associated transformations [*T*].

Step 1. Construct the list **G**_0_ of all possible integer 3-tuples, with each tuple element having a magnitude up to the maximum expected modulus *M*; typically **g** = (−5 ≤ *g*_0_ ≤ 5, −5 ≤ *g*_1_ ≤ 5, −5 ≤ *g*_2_ ≤ 5). Sort the list in order of increasing |**g**|, and omit **g** = (0, 0, 0).

Step 2. Construct **G**_1_, a copy of **G**_0_. Remove all items in **G**_1_ having 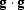 larger than a cutoff value, usually 6. Also delete tuples collinear to items higher on the list, *i.e.* 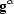 and 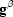 are collinear if 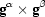 = 0. The remaining elements of **G**_1_ are possible values for **g** in equation (9). Note that the cutoff values *M* ≤ 5 and 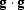 ≤ 6 are chosen empirically to permit the indexing of a large test set of diffraction images using the fewest computational cycles. These cutoffs give 37 **g** values in combination with three prime moduli (2, 3, 5) to give 111 formulae for reflection conditions.

Step 3. For each reflection condition [**g**, *M*] construct the matrix [*T*]. Begin by considering that every point on the reciprocal lattice obeys equation (9). In particular, the correct basis vectors will also follow this rule. Therefore, for the first row of [*T*] (the coefficients giving the correct basis vector a*) pick the first item 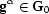 that satisfies 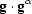 = *Mn*. Tuple 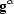 becomes the first row of [*T*]. For the second row of [*T*], select the first item 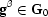 not collinear with 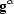 and satisfying the equation 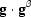 = *Mn*. Finally, the third row of [*T*] becomes the first item 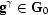 not coplanar with 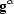 and 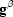, and satisfying the equation 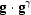 = *Mn*. If the determinant of [*T*] is negative, the first and second rows are switched.

In order to decide whether a basis set is primitive, each of the 111 reflection conditions enumerated in Step 2 is separately considered. Since the sample size of candidate Bragg spots is at most 600, the computational loop through all conditions is quite rapid (∼7 ms on a 2.8 GHz Intel Xeon processor). Allowing for a generous percentage of outliers (typically 20%), if the remaining spot candidates fulfill equation (9), a match is declared and the basis set is transformed with [*T*]. In Fig. 4 ▶(*b*), the correct basis set is immediately obtained from the matrix 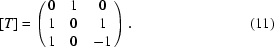
The loop is then repeated to search for any further systematic absences. Repetition of the loop allows us to limit the procedure to prime number moduli.

### 2.6. Selecting the best basis combination

The choice of basis vectors noted in equation (6) is not unique, since there are ∼20 directions in the set {**t**} to choose from. Certain combinations of directions may be immediately ruled out since they lead to unit cells with nearly zero volume. Specifically, if *V* is the cell volume and *a*, *b* and *c* are the cell axis lengths, a cutoff requirement that *V* > *abc*/100 does not lead to a significant loss of generality. For the remaining candidate bases, primitiveness is imposed (§2.5) and the basis choice is scored using a number of measures: (*a*) the root-mean-squared difference between **f** and **h**, where **f** and **h** are as defined in §2.4; (*b*) the number of candidate Bragg spots entering into the calculation of (*a*) after overlaps and axial spots are removed; (*c*) the root-mean-squared difference between observed and predicted laboratory coordinates of the candidate Bragg spots (r.m.s.d.); and (*d*) the fraction of candidate Bragg spots correctly predicted by the basis choice. Measures (*a*)–(*d*) provide the raw comparisons needed to pick a single high-scoring basis for all further work, with measures (*c*) and (*d*) being most useful.

The heuristic for choosing the basis was formulated empirically with the goal of producing a correct indexing solution in the least amount of computational time. With a well indexing crystal, many combinations of directions from {**t**} lead to similarly high scores, and give nearly identical unit-cell parameters. In such cases it is sufficient to try only a few combinations before making the final basis selection. With poor crystals it is often necessary to do an exhaustive search of possible basis vectors from {**t**} before choosing the best basis, or deciding that the diffraction pattern cannot be indexed. The actual method in the package represents an attempt to accommodate these two extremes, with the realisation that further fine-tuning may be beneficial. The implementation is presented as a high-level script in order to allow future changes or adaptations by the end user.

### 2.7. Cell reduction and refinement

For subsequent symmetry determination, it is necessary to calculate the transformation to a reduced basis as discussed in §9.3 of the *International Tables for Crystallography*, Vol. A (Burzlaff *et al.*, 1996 ▶). The essential requirement for the subsequent steps is that the reduced basis has vectors of minimum length. The reduced basis defined in the *International Tables for Crystallography* fulfills this requirement, but conventional iterative cell reduction algorithms leading to Buerger-reduced cells (Buerger, 1957 ▶; Gruber, 1973 ▶) or Niggli-reduced cells (Křivý & Gruber, 1976 ▶) are numerically unstable. A comprehensive treatment of this problem is given by Grosse-Kunstleve *et al.* (2004 ▶ *a*). Except where otherwise specified below, we adopted the minimum reduction presented in that work because it is fast and combines numerical stability with maximum portability.

After reduction, 12 model parameters are refined using conjugate-gradient minimization, with the minimization target being the root-mean-square difference (r.m.s.d.) between the observed and predicted Bragg spot positions introduced in §2.6. It should be emphasized that the target function includes only abstracted information about the positions of the ∼600 spots chosen for autoindexing; no information is present about pixel intensities on the original image. The first round of minimization adjusts the *x* and *y* coordinates of the direct beam, the next adds the crystal-to-detector distance, and the final round adds the nine components of the [*A*] matrix. First derivatives of the target function with respect to each parameter are calculated for the *LBFGS* minimization algorithm (Liu & Nocedal, 1989 ▶) implemented in our package *CCTBX* (Grosse-Kunstleve *et al.*, 2002 ▶). After minimization, the minimum reduction is applied again.

An estimate of the effective mosaic spread of the crystal is obtained separately. Diffraction patterns are calculated (Rossmann, 1979 ▶) using many trial values of mosaic spread ranging from 0 to 1.5°. The effective mosaic spread is taken to be the minimum value, which correctly predicts the observed positions of 80% of the ∼600 Bragg spot candidates used for indexing. The 80% requirement is chosen to allow a small fraction of outliers due to non-Bragg scattering or other pathologies. If the image is so poor that no value of mosaicity less than 1.5° will cover 80% of the spots, no further estimate is made.

### 2.8. Determination of the metric symmetry

With the diffraction pattern indexed, the final issue addressed is the crystal symmetry. Knowing the reduced basis, it is possible to find the highest possible Bravais symmetry consistent with its metric properties. Lower-symmetry lattices cannot be ruled out until the Bragg spot intensity data are scaled.

A fundamental concern here is that all data derived from experimental observations have some degree of uncertainty, leading to an imperfect knowledge of the reduced basis. Since lattice symmetry is by definition exact, any algorithm to deduce the Bravais type must necessarily use tolerances when testing symmetry conditions. We evaluated two distinct procedures against this criterion, ultimately adopting one of them.

In method A, the Niggli-reduced basis (Křivý & Gruber, 1976 ▶) is classified in terms of the 44 lattice characters listed in Table 9.3.1 of the *International Tables for Crystallography* (Burzlaff *et al.*, 1996 ▶). This approach tests elements of the metric tensor 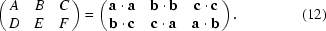
For example, one of the primitive tetragonal characters requires that (i) *A* = *B*, (ii) *D* = *E* = *F* = 0, and (iii) *DEF* ≤ 0. Equality tests such as (i) are evaluated within a tolerance parameter, set to 4% to accommodate normal levels of experimental uncertainty. For testing orthogonality conditions such as (ii) *D* = 0, similar reasoning implies that this expression should be considered true if the magnitude of the direction cosine between **b** and **c** is <0.04. It is more difficult to accommodate experimental uncertainty in evaluating condition (iii). If *D* = 0 or *E* = 0 or *F* = 0 (within 4% tolerance) then expression (iii) must be forced to be true even if *DEF* is numerically > 0. When necessary, it is possible to impose this condition by pre-multiplying two of the three basis vectors (**a**, **b**, **c**) by −1. While these procedures are adequate for most cells, we were able to identify cases where infinitesimal uncertainties in the basis vectors caused the Bravais type to be misidentified (§3.3).

We therefore adopted method B, in which the full Bravais symmetry is generated from a list of twofold rotational axes calculated from either the minimum-reduced or Niggli-reduced basis. Given the cell dimensions, the procedure of Le Page (1982 ▶) can identify a twofold axis by asking whether normal vectors to sets of real- and reciprocal-space planes coincide within a given angular tolerance δ. The tolerance is normally set to 1.4°, the minimum value needed to accommodate a wide number of test data sets. It is only necessary to consider a predefined list of 1379 pairs of normals to exhaustively find all candidate twofolds, and the code to implement this is fast and compact. Each discovered twofold is then converted to a matrix operator representation (Grosse-Kunstleve *et al.*, 2004*b* ▶). These symmetry operators are used as generators in a group-multiplication procedure to produce the complete symmetry group, which in turn is identified as one of the 14 Bravais types. An auxiliary procedure lists all possible subgroups. These are ranked by the maximum tolerance δ needed to accommodate all the twofold rotational axes of the subgroup. Transformations to standard settings are determined automatically according to Grosse-Kunstleve (1999 ▶).

### 2.9. Final restrained minimization

With the list of candidate Bravais settings, final parameter minimizations are performed (one for each candidate setting) to impose the metric conditions implied by the symmetry. Conjugate-gradient minimization is performed on the 12 parameters described above (§2.7), plus up to five additional restraints derived from symmetry. The derivation of these restraints is discussed in a separate paper (Grosse-Kunstleve *et al.*, 2004*b* ▶). At this point, it is sometimes possible to rule out the highest-symmetry candidate settings; for example, if the highest-symmetry setting produces a refined residual twice as high as that for the triclinic setting.

Indexing is now complete, with the final result being a set of parameters for each of the remaining candidate Bravais types. Optionally, these parameters may be converted into files suitable for *MOSFLM* (Leslie, 2001 ▶) input, so that Bragg reflections can be integrated and further analysis performed.

## 3. Validation of methods

### 3.1. Estimation of the direct-beam position is robust

It is well known that indexing relies critically on knowing the true position **m** at which the incident X-ray beam intersects the imaging detector. Although the beam coordinates can be refined to some degree after the indexing solution is found, the initial error Δ*m* in the position must be small enough to converge to the correct solution. The zone of convergence may be estimated by considering the unit-cell dimensions of the crystal. If the reduced basis vectors have lengths *a*, *b*, *c*, then the smallest spot-to-spot separation in the low-angle portion of the image will be of order *L* ≃ λ*D*/max(*a*, *b*, *c*), where λ is the X-ray wavelength and *D* is the crystal-to-detector distance. Clearly, if Δ*m* is of order *L* or higher, it will not be possible to index the diffraction pattern correctly.

To determine if the zone of convergence could be extended using the Fourier coefficient method, we considered a much larger region on the diffraction image, within a radius of 2.5*L* of the true beam center. In this experiment, a rectangular grid was superimposed onto this region, and each point **r** was separately treated as a prior-belief beam center, in a grid search for the true beam center **m** using the method outlined in §2.3. To determine if **r** lay within the zone of convergence, the true beam position was sought within a radius *S* = 1.3|**r** − **m**|. Determination of the new putative beam position was followed by indexing, least-squares parameter refinement, and Bravais lattice selection as outlined above (§§2.4–2.8 [Sec sec2.6] [Sec sec2.7] [Sec sec2.8]). The procedure was considered successful for a particular point **r** if it gave the correct beam center, Bravais lattice and unit cell. In a control experiment, we used the grid point **r** directly for the indexing step, relying only on subsequent least-squares parameter refinement to improve the beam position.

**Figure 5:**
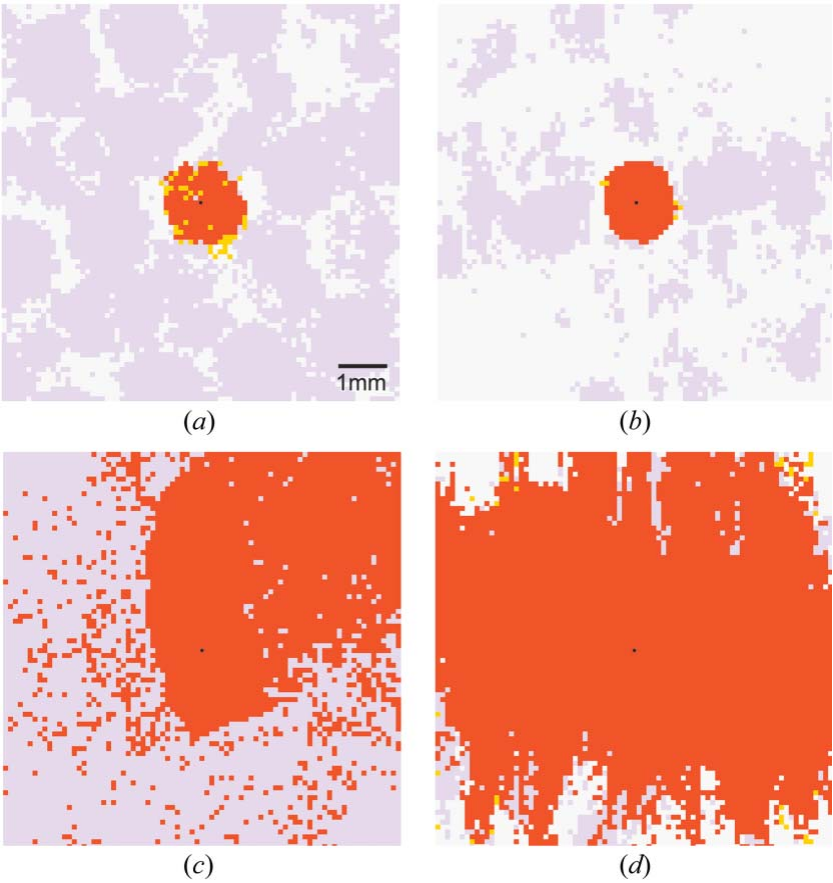
Ability to index the diffraction pattern as described in §3.1, given an input beam position at various coordinates on the detector. The portion of the detector shown is the same as in Fig. 3 ▶, with the true beam position shown as a black dot. The sample is the same as in Figs. 2 ▶ and 3 ▶, with the low-angle spot-to-spot separation from the longest cell dimension being **L** = 1.5 mm. Red pixels are input beam positions that lead to correct refined beam parameters, unit-cell dimensions and Bravais symmetry. Positions where otherwise correct indexing gives monoclinic symmetry (instead of orthorhombic) are given in yellow. Incorrect indexing is shown in lavender, and white pixels indicate that no indexing was possible. The first two panels show the control where the input beam position is used directly for indexing, with Bragg reflections from either one (*a*) or two (*b*) images. Note that in the single image used for panel (*a*), the **c** axis is parallel to the incident beam. Consequently, two lattice angles are poorly determined, accounting for the preponderance of input beam positions giving monoclinic rather than orthorhombic symmetry. When two images are used (*b*) this ambiguity disappears. The lattice-like arrangement of lavender patches in panel (*a*) corresponds to the lattice of probable (but incorrect) beam positions in Fig. 3(*c*) ▶. In the last two panels, the true beam position is predetermined by a grid search, based on one (*c*) or two (*d*) images.

A typical set of results is shown in Fig. 5 ▶. As expected, the control experiment produces a reliable solution only with an initial beam position error Δ*m* ≤ 0.4*L* (Figs. 5 ▶ *a* and 5 ▶ *b*). With the Fourier coefficient method, this particular image can be reliably indexed with any Δ*m* ≤ 0.6*L* (Fig. 5 ▶ *c*). The results improve even more dramatically when Bragg spots are combined from two images with ϕ settings 90° apart (Fig. 5 ▶ *d*). In this case, the correct solution is obtained whenever Δ*m* ≤ 1.2*L* = 1.8 mm. Note that to produce this result from the analysis of two images, one must assume that the detector remains stationary throughout the experiment. This is generally true for modern charge couple device (CCD) detectors and stationary phosphor imaging plates, but not for earlier detectors such as film or manually exchangeable imaging plates.

This simulation suggests that the beam position is likely to be discovered if Bragg spots are combined from two images and the search radius is set to *S* = *L*. Since the reduced basis is not available *a priori* to calculate *L*, it is reasonable instead to use the candidate cell lengths {*d*} corresponding to each direction in the set {**t**}. The search radius is set to 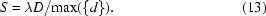
At present, knowing the beam center is generally considered to be a solved problem. Synchrotron beamlines, for example, often use a separate experiment to determine the beam coordinates before the crystal sample is exposed. However, there is a small but finite failure rate associated with such procedures. Invariably the final analysis requires visual inspection of the diffraction image, to confirm that the indexing solution agrees with the observed Bragg spots. If indexing fails, the correctness of the direct-beam position is typically the first item to be checked. In future applications, such as automated data collection, it will be necessary for indexing to occur automatically and nearly flawlessly, without time-consuming manual intervention. The Fourier coefficient method is attractive in this regard. It relaxes the stringency with which the beam center must be determined by other methods, and its built-in grid search effectively replaces the trial-and-error methods of visual inspection. It will be especially useful for crystals with large unit cells, where the indexing of closely spaced Bragg reflections is sensitive to small errors in the prior beam estimate.

In the preceding discussion, it is understood that the prior-belief beam position is obtained from information tabulated in the image file header, created at the time the data are acquired. A well known issue is that this tabulated position may be expressed in eight possible coordinate systems, with various detector manufacturers having chosen among the different conventions (Gewirth, 2003 ▶). It is important for the coordinate system to be properly identified prior to indexing, as it enters into the calculation of reciprocal-space coordinates **x**. Once this determination has been made, it is normally applicable to all images collected with a particular detector at a particular beamline.

### 3.2. Re-indexing solves a long-standing problem

Any procedure for deducing the lattice basis from experimental data must address the question of whether the resulting basis is primitive (Fig. 4 ▶). Caution is required because the candidate basis vectors in the list {**t**} are necessarily ranked by their ability to describe spatial periodicity in the data, that is, in order of highest |*F*(*l*)|, and not by shortest real-space vector length *d*. Yet it is imperative that the chosen vectors have minimal real-space length to assure that they are not linear combinations of the true basis vectors, as described in §2.5. Our test suite of 177 successfully indexed crystals provides numerous cautionary examples: in 90 of these samples, at least one combination of high-ranking candidate basis vectors [equation (6)] needs to be corrected by the procedure of §2.5, to assure proper indexing in the triclinic setting. Although this problem has long been alluded to in the literature (see for example §5.1 of Henry & Lonsdale, 1965 ▶), it has not, to our knowledge, been addressed directly by published procedures for indexing macromolecular diffraction data. In the paper by Steller *et al.* (1997 ▶), for example, basis vectors are chosen by the particular criterion of minimizing the number of reflections for which any component of **f** − **h** (§2.4) is greater than 0.2. We have found that using this criterion (or any of the other criteria suggested in §2.6) produces misindexing of the type illustrated in Fig. 4 ▶(*a*), in a small percentage of cases.

To compensate for this lack of adequate methods, macromolecular crystallography users have relied until now on interactive graphical indexing programs, which allow indexing by trial and error. If a basis set looks incorrect, program parameters such as the beam center and crystal-to-detector distance are slightly altered, which has the effect of producing a different basis choice. In the present context, however, we aim to index crystal lattices without the need for visual inspection of the result. The test presented in equation (9) permits automatic recognition of this important class of misindexed results, thus improving the reliability of any automated system.

### 3.3. Correct identification of rhombohedral symmetry by detection of twofolds

**Figure 6:**
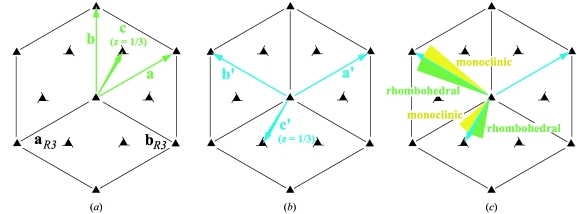
(*a*) Symmetry diagram for rhombohedral space group *R*3, showing the conventional 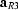 and 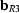 unit-cell axes in black. The 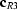 axis is perpendicular to the page. Case (i): axes (**a**, **b**, **c**) of the Niggli (reduced) cell shown in green, with the reduced **c** axis vector ending at a fractional position *z* = 1/3 above the plane of the page. (*b*) Case (ii), an alternate basis set [**a**′ = **a**, **b**′ = **b** − **a**, **c**′ = ** c** – 2(**a** + **b**)/3], equally likely to be chosen [equation (6)]. Cell reduction transforms (**a**′, **b**′, **c**′) back into the Niggli cell (**a**, **b**, **c**). (*c*) Mathematical perturbation or experimental uncertainty in the case (ii) **b**′ or **c**′ basis vectors breaks the symmetry, with the (**a**′, **b**′, **c**′) basis no longer reducing to (**a**, **b**, **c**) when either **b**′ or **c**′ is perturbed clockwise. If method A (§2.8) is then used to compute the metric symmetry, the result incorrectly depends on the sign of the perturbation.

We now examine an instance in which correct identification of the primitive basis can nevertheless lead to improper identification of the lattice symmetry, when method A (§2.8) is used for symmetry determination. Consider case (i), a hexagonal rhombohedral setting, with *a* = *b* = 10, *c* = 30, as depicted in Fig. 6 ▶(*a*), with the Niggli-reduced basis (**a**, **b**, **c**) as indicated. Using this basis to determine the metric symmetry of the unit cell, it can be shown algebraically that the metric tensor [equation (12)] satisfies these conditions: *A* = *B*; *D* = *E* = *F* = *A*/2. Looking for these conditions in Table 9.3.1 of the *International Tables for Crystallography* (Burzlaff *et al.*, 1996 ▶), one indeed discovers that they define character #9, one of four hexagonal rhombohedral characters. Note that since there is no prior information to bias the choice of basis, there is no guarantee that (**a**, **b**, **c**) will be selected as a candidate basis for the cell; in fact, it is equally likely that the vectors (**a**′, **b**′, **c**′) will be selected as shown in Fig. 6 ▶(*b*), case (ii). Although this basis is not reduced, application of the Křivý & Gruber (1976 ▶) algorithm immediately recovers the Niggli-reduced basis (**a**, **b**, **c**), and again the conditions are fulfilled for rhombohedral character #9.

Now we include experimental uncertainty in the basis vectors. Again considering case (i), beginning with basis (**a**, **b**, **c**) we can ask what will happen if vectors **b** or **c** are altered by small angular amounts. For either vector, a small perturbation does not alter the fact that the basis is in Niggli-reduced form. Method A (§2.8) allows a tolerance on the metric conditions, thus the finding that the likely lattice is of character #9 is unchanged. This is exactly as expected: a small uncertainty should not change the final conclusions about symmetry. In contrast, suppose that the starting point is the case (ii) basis set (**a**′, **b**′, **c**′), and consider perturbations of either **b**′ or **c**′ in the **ab** plane as shown in Fig. 6 ▶(*c*). Here the situation is quite different: application of the Křivý-Gruber algorithm gives the Niggli-reduced basis (**a**, **b**, **c**) when either **b**′ or **c**′ is rotated slightly counterclockwise, but a different Niggli-reduced basis when either is rotated clockwise. The alternate reduced basis satisfies different metric conditions, giving a monoclinic *C*-centered cell. Thus in case (ii), both experimental uncertainty and infinitesimally small theoretical perturbations can lead to misidentification of symmetry under method A.

It is appropriate to ask under what experimental conditions this can occur. We investigated the ability to determine the symmetry of a crystal in space group *R*32, with *a* = *b* = 143 Å, *c* = 519 Å. As explained in §2.6, the candidate basis (three directions) is selected from the set {**t**}, based on a ranking of how well the basis vectors can predict the input Bragg spots. Small differences in experimental parameters, particularly the direct-beam position, can influence the relative ranking of the candidate bases. We set up a two-dimensional grid around the true beam position, with each grid element in turn being considered as the assumed beam center. For the purpose of this test, no Fourier coefficients were used to re-determine the beam center (§2.3), nor was any parameter refinement undertaken; we simply took the highest scoring candidate basis, applied the Křivý-Gruber reduction, and attempted to determine the Bravais symmetry.

**Figure 7:**
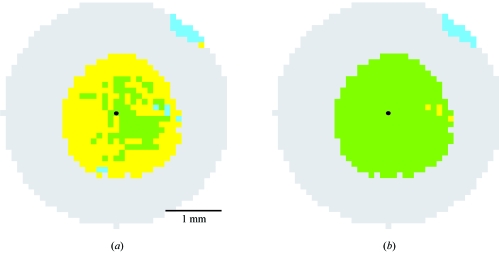
Determination of metric symmetry from a single oscillation image as a function of the input beam position. The true beam position is indicated by a black dot. Green pixels indicate correct determination of rhombohedral symmetry, while yellow denotes input parameters that incorrectly lead to a monoclinic *C*-centered lattice, and cyan denotes a triclinic lattice. Grey pixels indicate that no indexing is possible. Method A (§2.8) was used to determine symmetry in panel (*a*), while method B was used in panel (*b*).

Fig. 7 ▶(*a*) shows how well method A determines the symmetry when different grid points are assumed to be the beam center. The correct rhombohedral symmetry is deduced in fewer than half of the cases. Moreover, there is no zone of convergence for either the correct rhombohedral or the incorrect monoclinic symmetry; the symmetry determination oscillates in a chaotic manner as the imposed beam center moves across the image. Both symmetries can be deduced with input beam estimates within 0.1 mm of the true beam center. While Fig. 7 ▶(*a*) shows the results of indexing one 0.8° oscillation image, similar results were obtained for joint indexing of two images collected 90° apart in ϕ (not shown). In contrast, method B produces the correct solution across most of the grid (Fig. 7 ▶ *b*). These results support method B as a computationally tractable alternative that can unambiguously identify metric symmetry elements in the lattice. The method is thus well suited for inclusion in automatic systems.

## 4. Conclusions

While automated processing carries great potential benefit for the beamline user, it also places high demands for robustness upon its component algorithms and software. Problem areas that can be instantly recognized by the human experimentalist using a graphical interface may go unnoticed by an automated system, with potentially disastrous results for subsequent analysis steps. The methods presented above, if incorporated into the beamline control environment, will quickly produce reliable indexing and symmetry solutions immediately after the images have been acquired. A 2.8 GHz Intel Xeon processor typically requires 7 s to choose Bragg reflections from a pair of 10 Mbyte images, and 11 s for the remainder of the analysis.

It is particularly striking how a pair of images (Fig. 5 ▶ *d*) can yield a much more robust direct-beam position than a single image. It is also likely that the derived unit-cell dimensions are more accurate, since two images together sample reciprocal space more completely. With modern, scriptable beamline control software, it is typically easy to set up standard protocols to acquire these two images. One need only assume that the sample is rigidly affixed to the goniometer and is well centered in the beam. Thus it is important to exercise care when mounting and cryocooling the crystal.

We have successfully used *LABELIT* to process images from about 50 crystal forms. Notably, once the program parameters are switched to accept a high-resolution cutoff of 15 Å, *LABELIT* is able to index correctly tetragonal *I*-centered ribosome crystals with unit-cell dimensions *a* = *b* = 674 Å, *c* = 2776 Å (A. Vila-Sanjurjo & J. Cate, unpublished results). Therefore, the toolbox is expected to be generally applicable to all crystallographic experiments that use the oscillation method.

## 5. Program availability

*LABELIT* is organized as a hybrid software package in which the high-level scripts directing the algorithm use the Python scripting language, while the numerically intensive calculations are executed by optimized compiled C++ code. *LABELIT* makes extensive use of code objects from our open-source *Computational Crystallography Toolbox* (Grosse-Kunstleve *et al.*, 2002 ▶), and is therefore an example of the benefits of code reuse. Python bindings for C++ objects are provided by the Boost.Python library (Abrahams & Grosse-Kunstleve, 2003 ▶), and SCons (http://www.scons.org) is used as a build facility. *LABELIT* can be installed on a number of computing platforms, and customized for various data collection environments by modifying the included example scripts.

*LABELIT* will be available as a Web service at http://cci.lbl.gov/labelit, enabling general users to upload raw image data and retrieve the model parameters from the resulting indexing solutions. *LABELIT* will also be available for download to non-commercial users.

## References

[bb1] Abrahams, D. & Grosse-Kunstleve, R. W. (2003). *C/C++ Users J.* **21**, 29–36.

[bb2] Adams, P. D., Grosse-Kunstleve, R. W., Hung, L.-W., Ioerger, T. R., McCoy, A. J., Moriarty, N. W., Read, R. J., Sacchettini, J. C., Sauter, N. K. & Terwilliger, T. C. (2002). *Acta Cryst.* D**58**, 1948–1954. 10.1107/s0907444902016657 12393927

[bb3] Brunzelle, J. S., Shafaee, P., Yang, X., Weigand, S., Ren, Z. & Anderson, W. F. (2003). *Acta Cryst.* D**59**, 1138–1144. 10.1107/s0907444903008199 12832756

[bb4] Buerger, M. J. (1957). *Z. Kristallogr.* **109**, 42–60.

[bb5] Burzlaff, H., Zimmermann, H. & de Wolff, P. M. (1996). *International Tables for Crystallography*, Vol. A, *Space-Group Symmetry*, 4th revised ed., edited by T. Hahn, pp. 737–749. Dordrecht: Kluwer Academic Publishers.

[bb6] Cohen, A. E., Ellis, P. J., Miller, M. D., Deacon, A. M. & Phizackerley, R. P. (2002). *J. Appl. Cryst.* **35**, 720–726. 10.1107/s0021889802016709 PMC4041710 24899734

[bb7] Gewirth, D. (2003). *The HKL Manual*, 6th ed., p. 47. (http://www.hkl-xray.com.)

[bb8] Grosse-Kunstleve, R. W. (1999). *Acta Cryst.* A**55**, 383–395. 10.1107/s0108767398010186 10927267

[bb10] Grosse-Kunstleve, R. W., Sauter, N. K. & Adams, P. D. (2004*a*). *Acta Cryst.* A**60** In the press. 10.1107/s010876730302186x 14691322

[bb11] Grosse-Kunstleve, R. W., Sauter, N. K. & Adams, P. D. (2004*b*). *Acta Cryst.* A**60**, 1–6. 10.1107/s010876730302186x 14691322

[bb9] Grosse-Kunstleve, R. W., Sauter, N. K., Moriarty, N. W. & Adams, P. D. (2002). *J. Appl. Cryst.* **35**, 126–136.

[bb12] Gruber, B. (1973). *Acta Cryst.* A**29**, 433–440.

[bb13] Henry, N. F. M. & Lonsdale, K. (1965). *International Tables for X-ray Crystallography*, Vol. I, *Symmetry Groups*, 2nd ed. Birmingham: Kynoch Press.

[bb14] Kabsch, W. (2001). *International Tables for Crystallography*, Vol. F, *Crystallography of Biological Macromolecules*, edited by M. G. Rossmann & E. Arnold, pp. 218–225. Dordrecht: Kluwer Academic Publishers.

[bb15] Křivý, I. & Gruber, B. (1976). *Acta Cryst.* A**32**, 297–298.

[bb16] Le Page, Y. (1982). *J. Appl. Cryst.* **15**, 255–259.

[bb17] Leslie, A. G. W. (2001). *International Tables for Crystallography*, Vol. F, *Crystallography of Biological Macromolecules*, edited by M. G. Rossmann & E. Arnold, pp. 212–217. Dordrecht: Kluwer Academic Publishers.

[bb18] Leslie, A. G. W., Powell, H. R., Winter, G., Svensson, O., Spruce, D., McSweeney, S., Love, D., Kinder, S., Duke, E. & Nave, C. (2002). *Acta Cryst.* D**58**, 1924–1928. 10.1107/s0907444902016864 12393923

[bb19] Liu, D. C. & Nocedal, J. (1989). *Math. Programming*, **45**, 503–528.

[bb20] McPhillips, T. M., McPhillips, S. E., Chiu, H.-J., Cohen, A. E., Deacon, A. M., Ellis, P. J., Garman, E., Gonzalez, A., Sauter, N. K., Phizackerley, R. P., Soltis, S. M. & Kuhn, P. (2002). *J. Synchrotron Rad.* **9**, 401–406. 10.1107/s0909049502015170 12409628

[bb21] Otwinowki, Z. & Minor, W. (1997). *Methods Enzymol.* **276**, 307–326. 10.1016/S0076-6879(97)76066-X 27754618

[bb22] Pflugrath, J. W. (1997). *Methods Enzymol.* **276**, 286–306. 10.1016/S0076-6879(97)76065-8 27799101

[bb23] Pflugrath, J. W. (1999). *Acta Cryst.* D**55**, 1718–1725. 10.1107/s090744499900935x 10531521

[bb24] Rossmann, M. G. (1979). *J. Appl. Cryst.* **12**, 225–238.

[bb25] Snell, G., Cork, C., Nordmeyer, R., Cornell, E., Meigs, G., Yegian, D., Jaklevic, J., Jin, J., Stevens, R. C. & Earnest, T. (2004). *Structure*, **12**, 537–545. 10.1016/j.str.2004.03.011 15062077

[bb26] Steller, I., Bolotovsky, R. & Rossmann, M. G. (1997). *J. Appl. Cryst.* **30**, 1036–1040.

[bb27] Stevens, R. C., Yokoyama, S. & Wilson, I. A. (2001). *Science*, **294**, 89–92. 10.1126/science.1066011 11588249

[bb28] Zhang, Z., Sauter, N. K., van den Bedem, H., Snell, G. P. & Deacon, A. M. (2004). In preparation.

